# Interdomain Interactions Control Ca^2+^-Dependent Potentiation in the Cation Channel TRPV4

**DOI:** 10.1371/journal.pone.0010580

**Published:** 2010-05-11

**Authors:** Rainer Strotmann, Marcus Semtner, Frauke Kepura, Tim D. Plant, Torsten Schöneberg

**Affiliations:** 1 Institut für Biochemie, Medizinische Fakultät, Universität Leipzig, Leipzig, Germany; 2 Institut für Pharmakologie und Toxikologie, Fachbereich Medizin, Philipps-Universität Marburg, Marburg, Germany; University of Oldenburg, Germany

## Abstract

Several Ca^2+^-permeable channels, including the non-selective cation channel TRPV4, are subject to Ca^2+^-dependent facilitation. Although it has been clearly demonstrated in functional experiments that calmodulin (CaM) binding to intracellular domains of TRP channels is involved in this process, the molecular mechanism remains elusive. In this study, we provide experimental evidence for a comprehensive molecular model that explains Ca^2+^-dependent facilitation of TRPV4. In the resting state, an intracellular domain from the channel N terminus forms an autoinhibitory complex with a C-terminal domain that includes a high-affinity CaM binding site. CaM binding, secondary to rises in intracellular Ca^2+^, displaces the N-terminal domain which may then form a homologous interaction with an identical domain from a second subunit. This represents a novel potentiation mechanism that may also be relevant in other Ca^2+^-permeable channels.

## Introduction

Ca^2+^-dependent regulation by calmodulin (CaM) has been demonstrated for a large number of intracellular proteins including Ca^2+^-permeable plasma membrane cation channels. Often, this provides a negative feedback mechanism that either prevents excessive, potentially deleterious Ca^2+^ influx into the cell, or defines the time course of channel activity. Indeed, CaM-dependent inhibition is seen in most of the non-selective, Ca^2+^-permeable cation channels of the TRP group [1], [2], [3], [4], [5]. Within the TRPV subfamily, CaM-dependent regulation has been shown for the capsaicin and temperature-sensitive TRPV1 [3], [6], [7], temperature-sensitive TRPV3 [8], the highly Ca^2+^-selective epithelial Ca^2+^ channels TRPV5 and TRPV6 [4], [9], [10], and TRPV4 [11].

TRPV4 is highly expressed in kidney and was originally found to be activated by hypotonic solutions, suggesting a role in renal osmoregulation [12], [13], [14]. Studies thereafter identified multiple other stimuli (phorbol esters, temperature [15], [16], mechanical stimulation [17], [18], [19], [20], [21], [22] and arachidonic acid metabolites [23], [24]) and localizations suggesting diverse modes of activation in sensory and other systems. Like other TRP channels, TRPV4 is subject to dual Ca^2+^-dependent regulation with channel activity being potentiated and inactivated at Ca^2+^ concentrations attained during agonist-dependent activation [11], [25]. Ca^2+^-dependent facilitation has also been described for TRPC5 [26], TRPV3 [8], TRPV6 [9] and TRPA1 [27], [28].

In a previous study, we identified a CaM binding domain in the C terminus of TRPV4 as the structural basis of the Ca^2+^-dependent potentiation process [11]. Although CaM binding is a prerequisite for potentiation, the molecular mechanism that couples CaM binding to enhanced channel activity has not been resolved. Using protein interaction experiments and functional assays, we show that the molecular correlate of Ca^2+^-dependent current potentiation is disruption of an interdomain interaction within TRPV4 resulting from CaM binding to a C-terminal site. We provide experimental evidence for an underlying mechanism in which an N-terminal autoinhibitory domain forms a molecular switch that controls facilitation of TRPV4.

## Results and Discussion

### The C-terminal CaM binding site in TRPV4 binds to the CaM C lobe

Because CaM interaction with a C-terminal binding site in TRPV4 has been shown to be an essential step in the Ca^2+^-dependent potentiation of TRPV4 [11], we attempted to obtain initial clues about the underlying molecular mechanism by studying the CaM binding geometry. We analyzed the properties of the complex between CaM and C-terminal fragments of the human TRPV4 ortholog, and investigated the structural determinants within the CaM molecule that are involved in binding.

**Figure 1:**
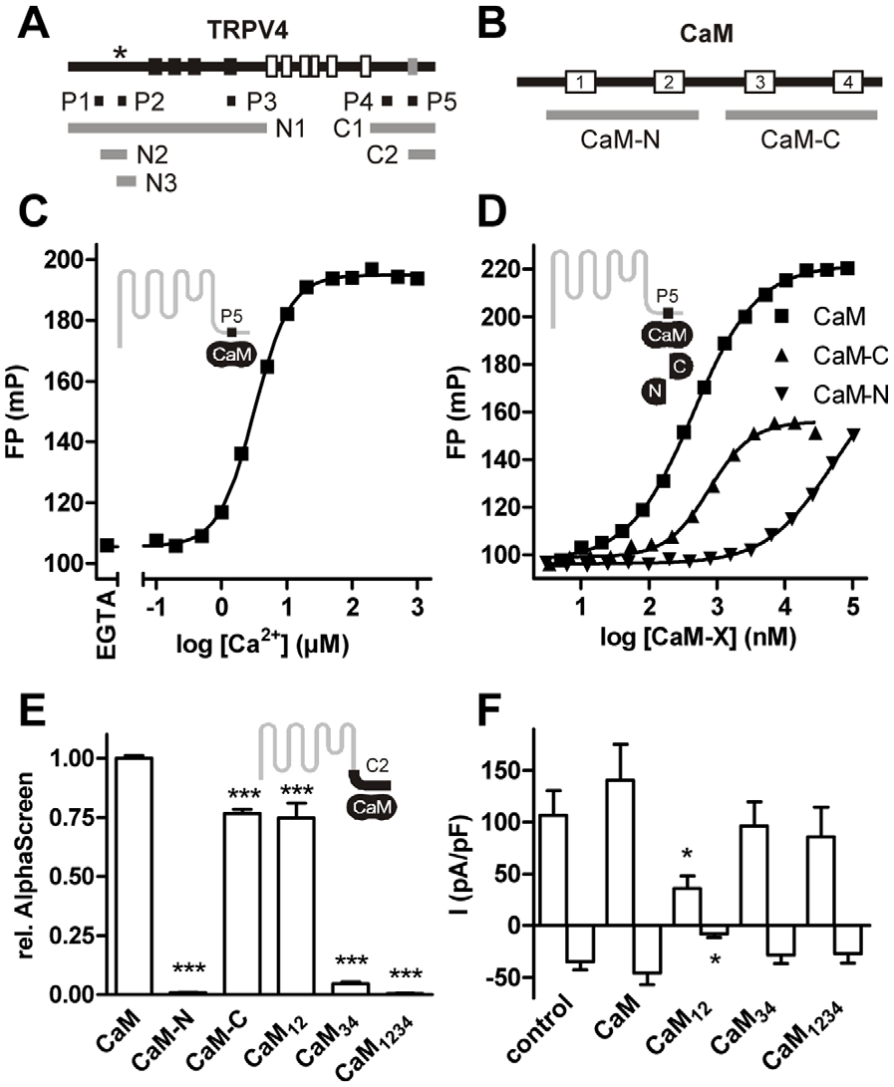
Lobe-specific CaM binding to TRPV4-C2. (A) TRPV4 topology. Ankyrin domains, transmembrane helices and the C-terminal CaM binding site associated with Ca^2+^-dependent potentiation are shown as black, white and grey boxes in the linearized TRPV4 structure. The positions of the protein fragments, peptides P1–5 and the N-terminal mutations (asterisk) used in this study are indicated below. For the precise positions see Table S1 (B) Structure of the CaM molecule. The EF hand domains 1 to 4 are shown as white boxes and the positions of the homologous lobe fragments used for the interaction experiments are indicated. (C) Ca^2+^-dependence of CaM binding to the fluorophore-labeled P5 peptide measured by fluorescence polarization. (D) Binding of the individual CaM lobes to fluorophore-labeled P5 peptide at 100 µM Ca^2+^. (E) Binding to the C2 fragment of the CaM lobes or CaM mutants that are lobe-specifically (CaM_12_, CaM_34_) or fully (CaM_1234_) Ca^2+^ binding-deficient (4 independent experiments, p≤0.001). (F) In HEK293 cells coexpressing TRPV4 and the indicated CaM mutants, whole-cell currents were activated by the application of 4α-PMA in Ca^2+^-free bath solutions. Current increases at +100 mV and −100 mV were measured after addition of 2 mM Ca^2+^ and compared to CaM overexpression (p = 0.013 and 0.007, respectively, for CaM_12_).

Fluorescence polarization experiments with the carboxyfluorescein-labeled CaM binding peptide, P5, as the tracer molecule (Fig. 1A, Table S1), showed that CaM binding was Ca^2+^-dependent and half-maximal at 3.2 µM Ca^2+^ (Fig. 1C). Thus, in contrast to some other TRP channels, where CaM binding is present at nanomolar Ca^2+^ concentrations [4], [29], CaM binding to TRPV4 depends on Ca^2+^ concentrations above those in resting cells. The Ca^2+^ concentration at which CaM binding to P5 was half-maximal is higher than the IC_50_ for steady-state inhibition of TRPV4 by intracellular Ca^2+^ in electrophysiological experiments (around 600 nM, [25]). However, during the dynamic response to an agonist, potentiation via this CaM binding site clearly precedes the inactivation process [11]. A possible explanation for the apparent discrepancy in the Ca^2+^ dependencies is that CaM binding to the full-length channel may occur at lower concentrations than to the short fragment used here. Alternatively, inactivation may be more sensitive to Ca^2+^, but a slower process than potentiation, or, in the case e.g. of Ca^2+^ entry through the channel, components of the potentiatory process may be exposed to a higher local Ca^2+^ concentration than those involved in inactivation.

**Figure 2:**
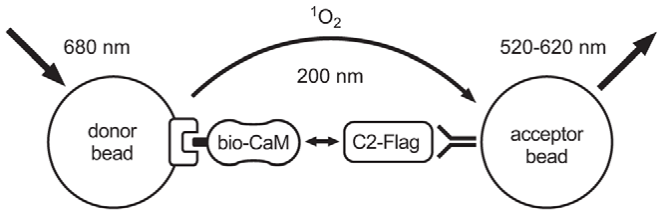
AlphaScreen-based CaM interaction assay. Optical stimulation of phthalocyanine compounds contained in the AlphaScreen donor beads excites ambient oxygen molecules into the metastable singlet state. The energy conveyed by the molecule is converted into light emission by the acceptor bead. Owing to the limited lifespan of singlet oxygen in solution, the AlphaScreen signal emission is highly dependent on the distance between the two beads. In this study, AlphaScreen donor and acceptor beads were modified for the detection of CaM/TRPV4 or interdomain interactions. Streptavidin-coated donor and anti-Flag acceptor beads were used to attach the proteins to the bead surface.

A characteristic feature of the CaM molecule is its symmetry with respect to its domain composition. The homologous N- and C-terminal lobes that both comprise two Ca^2+^-binding EF hand motifs (Fig. 1B), each expose a hydrophobic interaction domain on the molecular surface secondary to Ca^2+^ binding and may thus contribute independently to target binding. To quantify the individual affinities of the isolated CaM lobes to P5, we performed P5 fluorescence polarization experiments at different lobe concentrations in 100 µM Ca^2+^ (Fig. 1D). The Kd values were 400 nM, 700 nM and 25 µM for CaM and the CaM C-terminal (CaM-C) and N-terminal (CaM-N) lobes, respectively, indicating a 36-fold higher affinity of the CaM C lobe over the N lobe. In an independent experimental approach, we subjected a longer C-terminal TRPV4 fragment, C2 (Fig. 1A), to a CaM interaction assay that is based on the AlphaScreen proximity assay technology (Fig. 2, Fig. S1, Methods S1). Using different CaM constructs, we analyzed the relative binding affinities of the two CaM lobes alone or CaM mutants that are Ca^2+^ binding-deficient in the N-lobe (CaM_12_), C-lobe (CaM_34_) or both lobes (CaM_1234_) at 100 µM Ca^2+^ (Fig. 1E). Again, the interaction signal was found to be highly dependent on the presence of the functional C-lobe (Fig. 1E). The Ca^2+^-dependence of the interaction was similar for CaM, CaM_12_ and CaM_34_ with EC_50_ Ca^2+^ concentrations of 8.8±1.0 µM, 10.7±1.0 µM, 27.0±1.1 µM, respectively (Fig. S2).

To assess whether this finding is functionally relevant, we tested the effect of overexpression of CaM and CaM mutants on TRPV4 activity. In nominally Ca^2+^-free solutions, activation of TRPV4 by the phorbol ester 4α-PMA, a partial TRPV4 agonist [30]that we used in a previous study [11], is weak and slow. Addition of Ca^2+^ after activation by 4α-PMA results in facilitation followed by inactivation of the channel [11]. We measured the current increase on adding Ca^2+^ (2 mM) in HEK293 cells expressing YFP (not shown) or TRPV4 alone (control), or TRPV4 together with CaM or CaM mutants (Fig. 1F). CaM or the CaM mutants had no effect on spontaneous currents, or currents activated by 4α-PMA in a nominally Ca^2+^-free solution (not shown). A current increase upon addition of Ca^2+^ was observed in all cells except those expressing YFP alone. In the latter, currents were not significantly changed at +100 mV (difference: 0.133±0.438 pA/pF, n = 8, p = 0.7688), but significantly decreased at -100 mV (difference-1.097±0.436 pA/pF, n = 8, p = 0.04), probably due to a decrease in leakage current. CaM slightly increased the TRPV4 response to Ca^2+^, but the increase is not statistically significant, indicating that in HEK cells, the CaM concentration is not a limiting factor in TRPV4 potentiation. CaM mutants with a dysfunctional C lobe (CaM_34_, CaM_1234_) had no effect (Fig. 1F), probably because these mutants do not interact with the TRPV4 C-terminal domain and do not functionally compete with endogenous CaM. In contrast, current increases were significantly smaller with the mutant CaM_12_ that binds to TRPV4. This mutant may bind and displace endogenous CaM, but, owing to the N-terminal mutation, cannot fully substitute for the wild type molecule. This suggests that, in spite of its lower affinity to the C-terminal CaM binding site in TRPV4, the CaM N lobe contributes functionally to TRPV4 potentiation by a mechanism which remains unclear.

These results with CaM mutants differ from those on Ca^2+^-dependent potentiation in other TRP channels. Coexpression of CaM_1234_ abolished TRPC6 activation secondary to G_q_ protein-coupled receptor activation or diacylglycerol application [31], [32] and strongly decreased activation of the adenosine diphosphoribose-sensitive channel TRPM2 [33], [34]. For TRPM2, co-immunoprecipitation experiments demonstrated CaM_1234_ binding to the channel, indicating that Ca^2+^-independent CaM binding contributes to the facilitation mechanism. In contrast to these findings, TRPV4 neither binds to CaM in Ca^2+^-free buffers, nor to CaM_1234_, and potentiation of its activity directly depends on CaM interaction triggered by a rise in Ca^2+^.

How does CaM binding facilitate Ca^2+^ entry through TRPV4 at the molecular level? Two general molecular switching mechanisms seemed conceivable. The first comprises a ternary interaction in which Ca^2+^-loaded CaM binds to two different sites within the TRPV4 protein thus bringing two regulatory domains in proximity to each other. Indeed, asymmetrical CaM binding has been demonstrated for the pore-forming subunits of the CaV1/CaV2 [35], [36] and SK [37], [38] channel families, and a similar mechanism has been proposed for the Ca^2+^-dependent desensitization of TRPV1 [6], [7]. Using CaM overlay experiments on a peptide library representing the cytosolic termini of TRPV4 and fluorescence polarization experiments we identified three additional CaM binding sites in TRPV4 that may serve as anchoring domains for the CaM N lobe (Fig. S3). However, in fluorescence polarization experiments, their CaM affinities were in the micromolar range (Fig. S4) and none bound to CaM-N with higher affinity than to CaM-C (Table S2).

### CaM binding disrupts an interdomain interaction

The alternative scenario for CaM-dependent current facilitation in TRPV4 involves a disinhibition process in which CaM binding displaces a regulatory domain that is attached to TRPV4 in the resting state. Prototypical mechanisms involving the displacement of autoinhibitory domains from a functional center are found in the CaM-dependent activation of CaMK II [39] and the plasma membrane Ca^2+^ pump [40], [41]. Multiple lines of evidence indicate that this is indeed the mechanism of the CaM-dependent potentiation in TRPV4.

**Figure 3:**
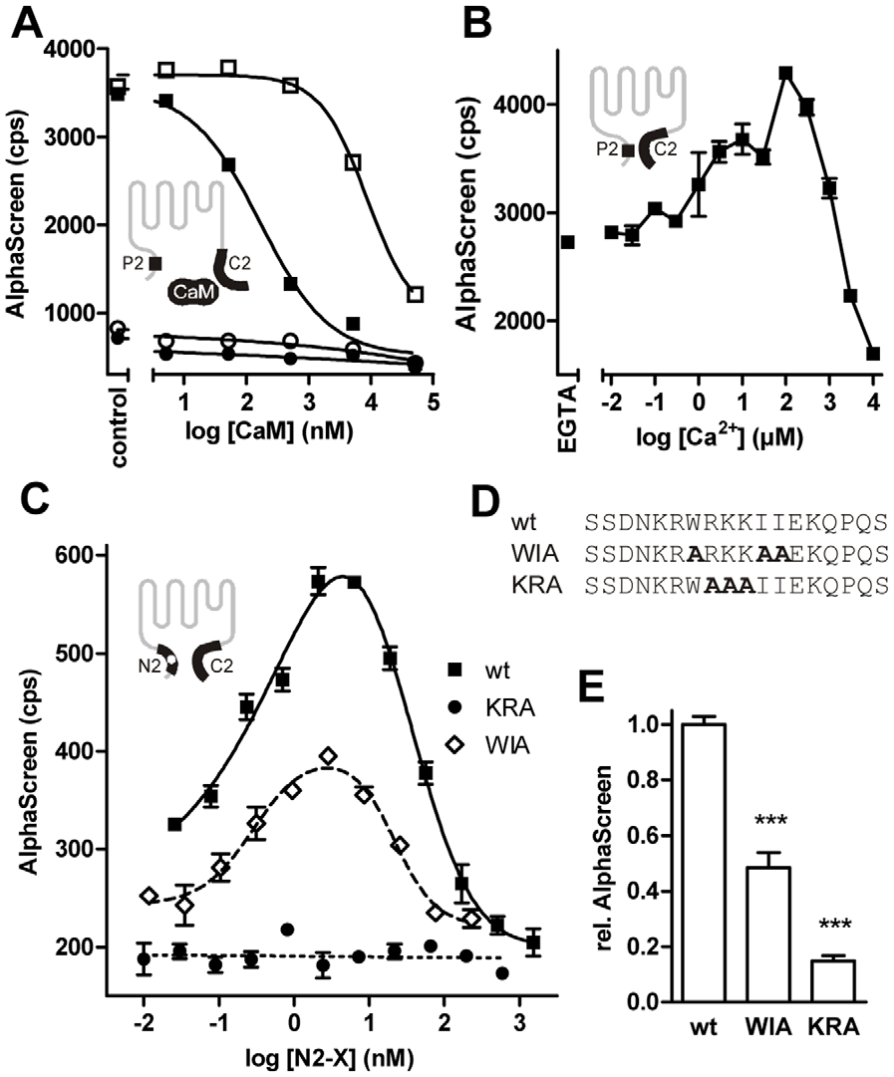
The C2 fragment interacts with an N-terminal domain. (A) P2 and CaM bind competitively to C2. C2-Flag interaction with P2-biotin (squares) or, as control protein, C2-biotin (circles) was measured in an AlphaScreen interaction assay in the presence of CaM at the indicated concentrations or in the absence of CaM (control). Buffers contained either 100 µM Ca^2+^ (filled symbols) or 1 mM EGTA (open symbols). A strong constitutive interaction between C2 and P2 was observed that was inhibited by Ca^2+^/CaM. (B) The C2-P2 interaction is Ca^2+^-independent over a wide range of Ca^2+^ concentrations. (C and D) Mutation of specific amino acids in the N-terminal binding domain abolishes the interdomain interaction. Binding of C2-biotin to the N2-Flag (wt) or the mutants N2-WIA and N2-KRA given in D was measured at concentrations between 10 pM and 1 µM in buffers containing 100 µM Ca^2+^. (E) Curve maxima from C were tested against wild type. Both mutants showed significantly lower interaction signals (p<0.0001, 5 independent experiments).

First, we did a complementation experiment to test whether a CaM molecule that binds to the C2 fragment of TRPV4 through its C-lobe can form an additional interaction with another CaM binding domain thus forming a ternary complex. We focused on the N-terminal P2 peptide which with its accumulation of positively charged amino acids around a central tryptophan residue resembles the CaM-binding M13 peptide from the skeletal muscle myosin light chain kinase, a prototypical CaM binding motif. We used an AlphaScreen-based proximity assay (Fig. 2, Methods S1) in which the acceptor beads were coated with the C-terminal fragment C2-Flag, and the donor beads either with the N-terminal CaM binding peptide P2-biotin, or, as a negative control, with C2-biotin. The interaction between the proteins was measured in the presence of increasing CaM concentrations in 100 µM Ca^2+^ or 1 mM EGTA (Fig. 3A). Surprisingly, instead of CaM-dependent complex formation, a strong constitutive interaction between the N- and C-terminal TRPV4 fragments was observed in the absence of CaM (Fig. 3A). CaM competitively reduced this interaction in a Ca^2+^-dependent fashion with an IC_50_ of 166 nM in 100 µM Ca^2+^, a value that is in agreement with the Kd observed for its binding to C2 (see Fig. 1D). The control reaction in which both acceptor and donor beads were coated with the C2 protein did not show interaction in the absence or presence of CaM (Fig. 3A).

To determine the Ca^2+^-dependence of the complex between P2 and C2, an AlphaScreen experiment was performed in the absence of CaM but with Ca^2+^ concentrations between 10 nM and 10 mM (Fig. 3B). The interaction was already present at 0 Ca^2+^ and essentially Ca^2+^-insensitive over a wide range of Ca^2+^ concentrations. Very high Ca^2+^ concentrations above 100 µM, however, inhibited binding of the fragments, a process that is probably secondary to unspecific Ca^2+^ binding and has been observed for other protein interactions including CaM binding to TRPV4 [11].

To more finely map the interaction site, we then performed overlay experiments with a partial peptide library from the TRPV4 N terminus (Fig. S3A). Probing with the C2 fragment resulted in strong binding to a site that overlaps with the P2 peptide. In a similar fashion, a peptide array from the TRPV4 C terminus was probed with the N-terminal N3 fragment (Fig. 1A) that includes P2 (Fig. S3B). Consistent with the finding that CaM inhibits the interaction, strong interaction was found with a region that corresponds to the CaM binding peptide P5.

The interdomain interaction could also be shown using the longer N-terminal TRPV4 fragments N2 (wt in Fig. 3C) and N3 (data not shown) instead of P2. In an AlphaScreen interaction experiment, donor and acceptor beads were incubated with 10 nM C2-biotin and N2-Flag at concentrations ranging from 30 pM to 1.5 µM, respectively (Fig. 3C). Increasing N2-Flag concentrations resulted in increasing C2 interaction signals with a maximum at the bead capacity of approximately 10 nM bound protein. Further increases in the N2-Flag concentration led to signal reduction owing to a hook effect of the assay system, demonstrating specific interaction of the fragments (see Methods S1).

Secondly, N2 mutants in which key basic amino acids within the N-terminal binding motif were changed into alanine showed significantly weaker binding to C2. We used site-directed mutagenesis to create N2 mutants that lack either the hydrophobic (WIA) or the positively charged residues (KRA) in the N-terminal interaction domain (Fig. 3D). In AlphaScreen-based C2 interaction assays, both constructs, WIA and KRA, had a significantly lower binding affinity to the C2 fragment (Fig. 3C,E) with respective AlphaScreen signals of 48.4±5.5% and 14.8±2.1% of the wild type at the given component concentrations.

**Figure 4:**
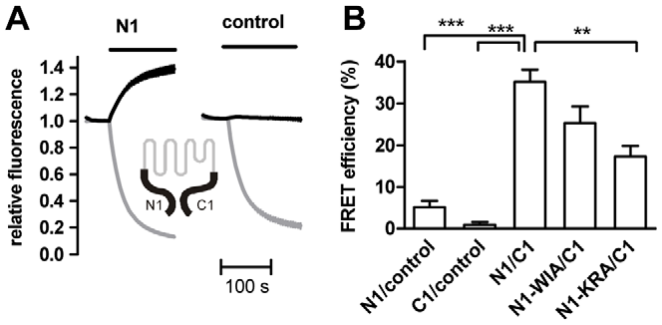
The TRPV4 N and C termini interact in HEK293 cells. (A) YFP-fused C1 fragment and CFP-fused N1 (left) or CFP (right) were coexpressed in HEK293 cells and the donor and acceptor fluorescences (black and grey traces, respectively) measured during selective YFP photobleaching (bar, 6 and 3 independent experiments). (B) FRET acceptor bleaching measurements as shown in A were performed with the indicated donor/acceptor combinations. YFP and CFP, respectively, were used as acceptor and donor controls (3 independent experiments).

The same was found when CFP- and YFP- fusion protein of the full-length N and C-termini, respectively, (N1, C1, Fig. 1A) were tested for interaction in fluorescence resonance energy transfer (FRET) experiments after coexpression in HEK293 cells (Fig. 4). This is in agreement with a recent study [42] that showed high FRET efficiency in TRPV4 for both homologous N-terminal and N-terminal to C-terminal interactions. Interestingly, the interaction site represented by the P2 peptide is close to a proline-rich domain that has been shown to interact with PACSIN 3, a regulatory protein that controls basal TRPV4 activity and stimulus specificity [43], [44].

**Figure 5:**
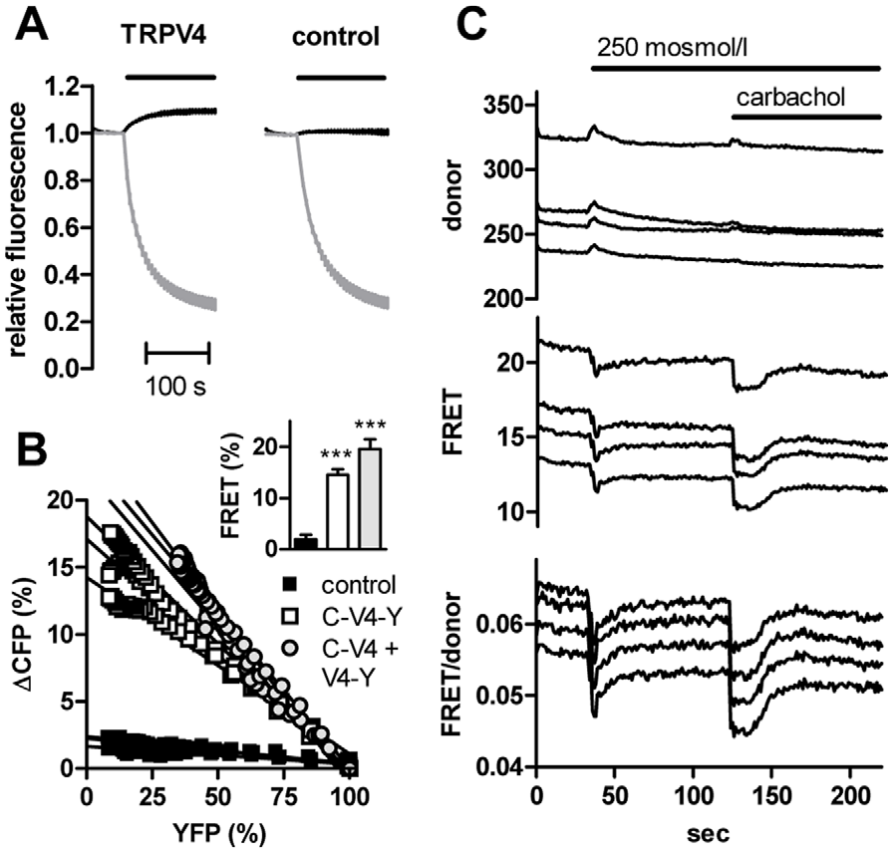
Calcium-dependent interdomain interaction in TRPV4. (A) A CFP-TRPV4-YFP fusion construct (TRPV4) or TRPV4-YFP cotransfected with CFP (control) were subjected to FRET acceptor bleaching experiments after expression in HEK293 cells. (B) FRET efficiencies were calculated by the extrapolated donor increase at full acceptor photobleaching (three cells shown from each group). The FRET efficiencies from the experiments shown in (A) (labeled C-V4-Y and control), and cells cotransfected with CFP-TRPV4 and TRPV4-YFP (labeled C−V4+V4−Y), were 14.6±1.1, 2.0±0.8 and 19.6±1.9. Both CFP-TRPV4-YFP and CFP-TRPV4+TRPV4-YFP interactions were significantly different from control (inset, p<0.0001, n = 7, 20 and 13, respectively). (C) The donor and FRET channels were recorded in HEK293 cells expressing CFP-TRPV4-YFP during application of hypotonic medium and 10 µM carbachol.

To investigate the interaction in the full-length channel protein, we used a TRPV4 expression construct in which CFP and YFP were fused to the N- and C termini, respectively (Fig. 5). Fluorescence resonance energy transfer (FRET) experiments in unstimulated HEK293 cells expressing the construct revealed a significantly stronger FRET efficiency between the fluorophores than in cells cotransfected with a TRPV4-CFP fusion construct and YFP (Fig. 5A,B). The same was found in cells cotransfected with CFP-TRPV4 and TRPV4-YFP (Fig. 5B) suggesting that interactions can occur between subunits in the homotetrameric channel. All constructs resulted in functional channels (data not shown). Interestingly, both stimulation of CFP-TRPV4-YFP by decreases in the extracellular osmolarity (Fig. 5C) or application of 4α-PMA (not shown) in Ca^2+^-containing buffer, and intracellular Ca^2+^ release after stimulation of the endogenous M3 muscarinic receptor resulted in transient decreases in the FRET- and concomitant increases in the donor channel fluorescences, indicating a decrease in FRET efficiency (Fig. 5C).

### Inhibition of the interaction between N and C termini renders TRPV4 permanently potentiated

**Figure 6:**
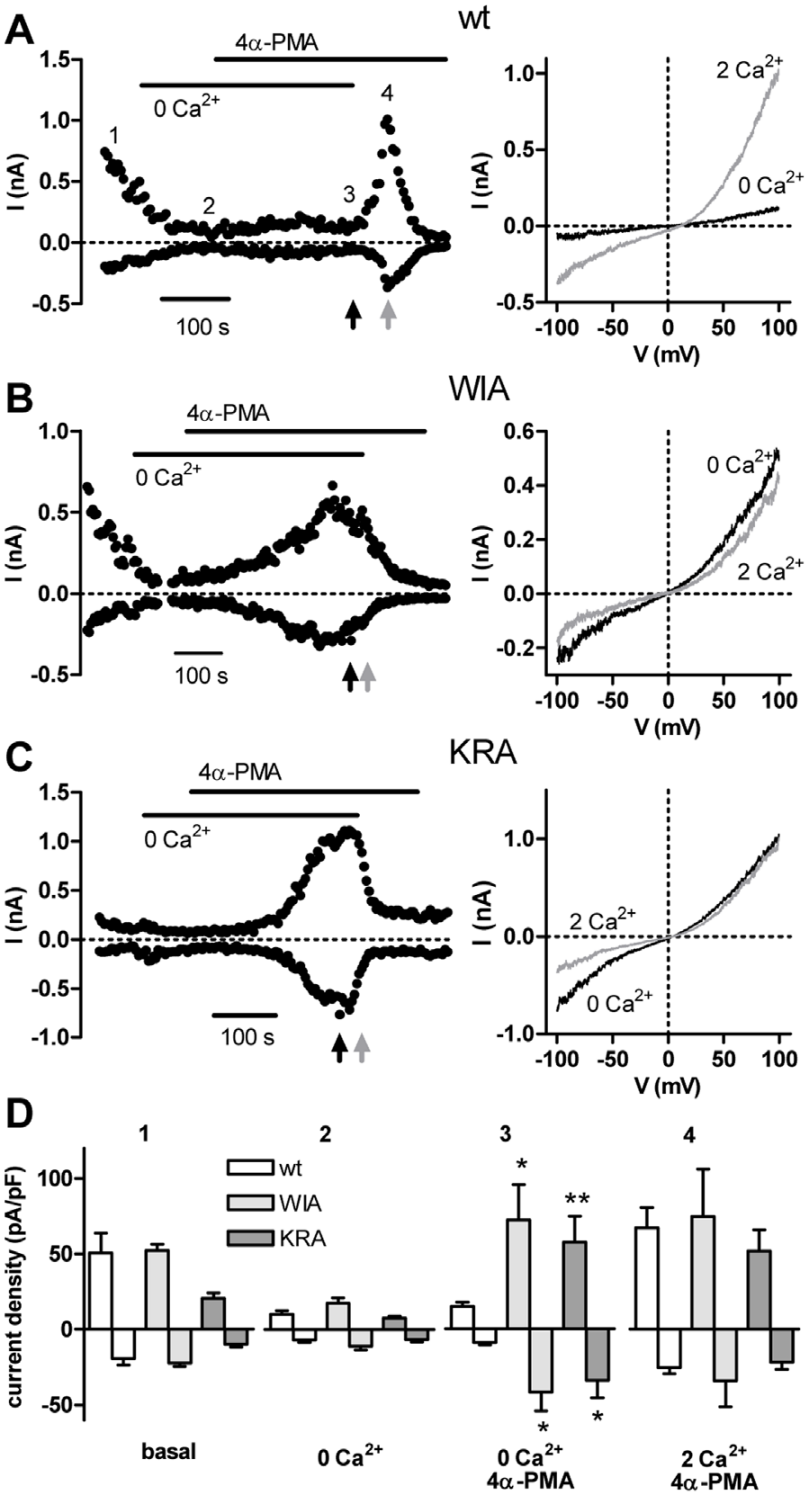
TRPV4 mutations that disrupt the interaction between the N- and C-terminal domains result in channels that are strongly activated in the absence of extracellular Ca^2+^. (A–C) Time courses (left) of currents at -100 and +100 mV showing the activation by 4α-PMA (1 µM) in a nominally Ca^2+^-free solution (0 Ca^2+^) and the effect of readdition of 2 mM Ca^2+^ for wt TRPV4 (A) and the mutants WIA (B) and KRA (C). The IV-relationships (right) were recorded in 4α-PMA just before and shortly after switching to 2 mM Ca^2+^ as indicated by the arrows. (D) Mean current densities at +100 and −100 mV measured after break-in (basal) and at the start of 4α-PMA application in a nominally Ca^2+^-free solution (0 Ca^2+^), and the mean maximum inward and outward current densities attained in 4α-PMA in the nominally Ca^2+^-free solution, and following the addition of 2 mM Ca^2+^ in 4α-PMA (see numbers in A). The currents measured after stimulation in nominally Ca^2+^-free solutions at -100 and +100 mV for the WIA (p = 0.014 and 0.023) and KRA mutants (p = 0.012 and 0.007) were significantly larger than the wild type (n = 9, 8 and 5 for wt, WIA and KRA).

As a third line of evidence, the full-length WIA and KRA mutants displayed significantly enhanced currents during activation in nominally Ca^2+^-free media (Fig. 6). In whole-cell patch-clamp experiments, TRPV4 wild type showed an initial decrease in spontaneous activity after establishment of the whole cell configuration (Fig. 6A). Addition of 4α-PMA thereafter in the nominal absence of Ca^2+^ led to a slow, small increase in currents which after reaching a plateau were then strongly, but transiently potentiated on readdition of Ca^2+^. Potentiation was rapidly followed by Ca^2+^-dependent inhibition. Both mutants showed clear differences to wild type TRPV4. Significantly larger currents were activated by 4α-PMA in the nominally Ca^2+^-free medium and no potentiation, only inhibition was observed on readdition of Ca^2+^ (Fig. 6B, C). Interestingly, the current densities attained by the mutants in 4α-PMA were similar to those for wild type TRPV4 after potentiation by Ca^2+^. Thus, channels with the mutations that prevent or reduce interactions between the N and C termini do not require increases in intracellular Ca^2+^ for strong current activation.

All data provide strong evidence for an interaction in TRPV4 between an N-terminal domain represented by the P2 peptide and a site in the C terminus, P5, that also binds to CaM. Our functional data show that disruption of the interaction by mutagenesis of key amino acids leads to constitutive current potentiation when the channel is stimulated in the absence of extracellular Ca^2+^. This is in good agreement with a previous study [11] that demonstrated that TRPV4 mutants in which CaM binding to the C terminus is abolished result in channels not potentiated by Ca^2+^ addition.

A similar interdomain interaction mechanism has also been found in cyclic nucleotide-gated channels [45]. Here, an N-terminal domain is capable of interacting with both the cyclic nucleotide binding region in the C terminus of the protein and Ca^2+^/CaM. In contrast to TRPV4, however, this is functionally coupled to autoregulatory channel inactivation. The same group demonstrated using GFP-FRET experiments that the interaction occurs between adjacent subunits of the tetrameric protein rather than within the same subunit [46].

### The N-terminal interaction domain is capable of dimerization

**Figure 7:**
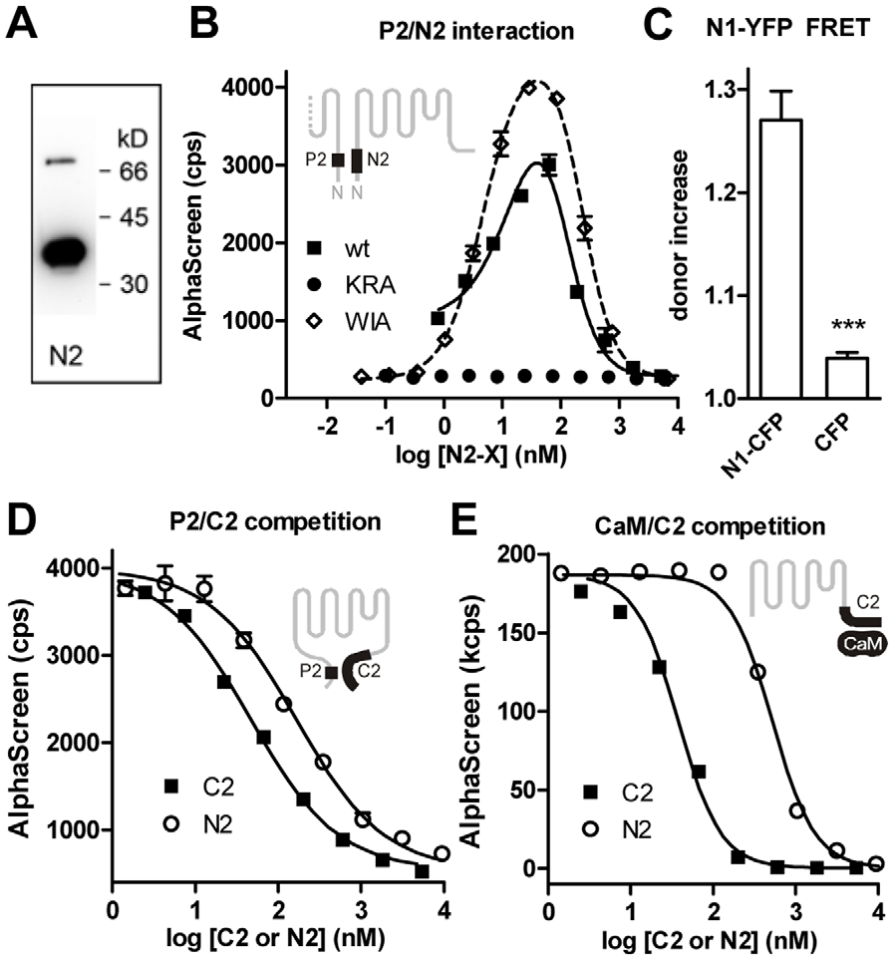
The P2 peptide contributes to homodimerization of the TRPV4 N terminus. (A) The purified and biotinylated N2-GST protein was subjected to SDS-PAGE, blotted and visualized in avidin-peroxidase overlay experiments. In addition to the monomeric form at 34.8 kD, a second band at a size consistent with that of the N2 dimer was detected. (B) The P2 peptide interacts with the wild type N2 fragment and the WIA but not the KRA mutant. In an AlphaScreen experiment, biotinylated P2 peptide was subjected to interaction with wild type N2 fragment (wt) or the mutants WIA or KRA at the indicated concentrations. (C) The full-length TRPV4 N termini show dimerization in FRET acceptor bleaching experiments. (p<0.0001, 4 and 3 independent experiments). (D) The interaction between P2-biotin and C2-Flag is inhibited by the C2 fragment with a 3.5-fold lower IC_50_ than the N2 fragment. (E) The complex between CaM-biotin and C2-Flag at 100 µM Ca^2+^ is inhibited by C2 with a 14-fold higher affinity than by N2.

When the chromatographically purified N2 protein was subjected to SDS-PAGE, a strong band of the expected size of 34.8 kD was detected (Fig. 7A). In addition, a second band was visible with a molecular weight compatible with a dimeric form of N2 (Fig. 7A). In gel filtration experiments under non-denaturing conditions, a single peak was detected at a size that corresponds to the dimeric form (Fig. S5). We then used an AlphaScreen-based assay to investigate the interaction of the N-terminal P2 peptide with the wild type N2 fragment or the N2 mutants WIA and KRA in nominally Ca^2+^-free buffers (Fig. 7B). P2 bound strongly to wild type N2 and the WIA mutant, but not to the KRA mutant. Similar results were obtained using the biotinylated N2 fragment instead of the P2 peptide (data not shown). These data show that the N-terminal domain constituted by the P2 peptide, in addition to its affinity for the C-terminal binding site, has the ability to form homodimeric interactions.

FRET experiments were performed to validate the interaction under intracellular conditions (Fig. 7C). N1-YFP was coexpressed with N1-CFP or CFP alone in HEK293 cells and the donor fluorescence measured during selective YFP photobleaching. The respective maximal relative donor fluorescence increases after acceptor bleaching were 1.27±0.03 and 1.04±0.01. We conclude that there is significant interaction between two molecules of the full-length TRPV4 N terminus that is dependent on the integrity of the domain formed by the P2 peptide.

It is known that TRPV4 forms homotetrameric complexes [47]. The N termini of adjacent subunits are thus readily accessible for homologous interaction. Indeed, previous studies [42] demonstrated that strong binding between the N-terminal tails exists in TRPV4. Studies in TRPV4 [48], TRPV5 [49] and TRPV6 [50] indicated that specific N-terminal ankyrin domains are crucial structures for channel self assembly. However, the N2 fragment that includes the P2 peptide is located N-terminal to the ankyrin domains (Fig. 1A). The dual affinity of this domain to both a homologous domain of a second subunit and the C-terminal CaM binding site suggests that it may contribute to transient rather than static interactions in the functional channel.

### Dual role of the N-terminal interaction domain

To investigate which of the alternative interactions of the P2 peptide is relevant in the absence and presence of CaM binding to the TRPV4 C terminus, we performed a series of competition experiments (Fig. 7D,E). AlphaScreen acceptor and donor beads were coated with C2-Flag and either P2-biotin (Fig. 7D) or CaM-biotin at 100 µM Ca^2+^ (Fig. 7E) and incubated with untagged C2 or N2 competitor proteins at the indicated concentrations. In the P2/C2 complex (Fig. 7D), the IC_50_ values for C2 and N2 were 47 nM and 166 nM, respectively, indicating a 3.5-fold higher apparent affinity of the C2 fragment. A similar finding was observed when the CaM/C2 interaction was subjected to competition by C2 or N2 (Fig. 7E). The IC_50_ values for C2 and N2 were 38 nM and 522 nM. Taken together, the data indicate a higher affinity of the N-terminal binding site towards C2 than to a second N-terminal fragment, suggesting that in the resting state of the channel when CaM is not bound to the C-terminal binding site, the interaction between the N and C termini prevails.

**Figure 8:**
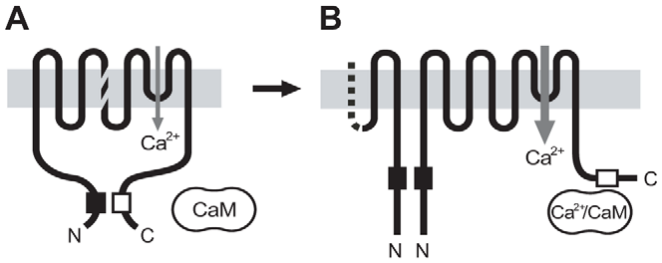
Proposed TRPV4 potentiation mechanism. (A) In the resting state, an N-terminal site (filled box) binds to a C-terminal domain (open box) thus permitting only weak channel activation. The hatched lines in the fourth transmembrane region are meant to indicate that the interaction can occur between channel subunits or possibly within a channel subunit. (B) Increase of the intracellular calcium concentration to micromolar concentrations leads to Ca^2+^/CaM binding to the C-terminal site and displacement of the N-terminal interaction site. The corresponding conformational change induces further increase in channel activity. In the potentiated state, the N-terminal binding site may form a homologous interaction with a second channel subunit.

In summary, this study provides a comprehensive model for the Ca^2+^-dependent potentiation of TRPV4 currents. Using different experimental strategies, we have demonstrated that an interaction between an N-terminal and a C-terminal site in TRPV4 is present at nanomolar Ca^2+^ concentrations (Fig. 8A), but becomes disrupted secondary to CaM binding to the TRPV4 C terminus at micromolar Ca^2+^ concentrations (Fig. 8B). In *in vitro* FRET experiments, the interaction was shown to occur between channel subunits, presumably in the channel homotetramer. However, from the experimental data, we cannot exclude the possibility that the N and C termini of a single channel subunit can interact.

Evidence for an interaction between N and C termini was also obtained in functional experiments. While TRPV4 mutants that are CaM binding-deficient result in currents that are small and do not potentiate secondary to rises in the intracellular Ca^2+^ concentration [11], mutants in which the interaction is absent result in large currents in the absence of Ca^2+^ (Fig. 6). A second, homologous interaction between the N termini of adjacent TRPV4 subunits that is present when CaM is bound to the C-terminal site may contribute to conformational changes that lead to current facilitation (Fig. 8B), but we currently have no functional evidence in support of this. This model represents a new Ca^2+^-dependent regulatory mechanism that may also be found in other CaM-regulated cation channels.

## Materials and Methods

### AlphaScreen protein interaction measurements

Proteins were expressed in *E. coli*, affinity purified and biotinylated if applicable (for details see Methods S1). For protein interaction measurements, a proximity assay system based on the AlphaScreen technology (PerkinElmer) was used (see Methods S1). Briefly, streptavidin-coated AlphaScreen donor and anti-Flag acceptor beads at a final concentration of 16.7 µg/ml were incubated in a 384-well plate format (OptiPlate-384, PerkinElmer) with C2-GST-biotin or P2-biotin and the respective Flag-tagged interaction protein at concentrations of 10 nM, if not otherwise indicated. Assay buffer conditions were 50 mM Tris, pH 7.4, 100 mM KCl, and 0.1% BSA. After incubation for 90 min at room temperature, the AlphaScreen output signal was recorded in a Fusion alpha FP instrument (PerkinElmer) at 2 s reading time per well. AlphaScreen signals are given as mean±SEM in counts per second (cps) units.

### Fluorescence polarization

Tracer peptides were synthesized and coupled to carboxyfluorescein (Dr. Sven Rothemund, Core unit peptide technologies, IZKF, Leipzig). Protein interaction experiments were performed in buffers containing 50 mM Tris, pH 7.5, 100 mM KCl, 0.1% BSA and calcium at the indicated concentrations. Calcium concentrations below 50 µM were buffered with HEDTA, EGTA or EDTA at a buffer concentration of 10 mM. CaM concentrations were 10 µM or as indicated; the tracer peptide concentration was 100 nM. Fluorescence anisotropy was measured in a 384-well black multititer plate (Fluotrac 200, Greiner) using a Fusion alpha FP instrument (PerkinElmer) at 1 s reading time per well. Fluorescence polarization (FP) was calculated according to:
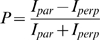
with I_par_ and I_perp_ as the fluorescence emission intensities in planes parallel and perpendicular to the excitation plane. FP signals are given as mean±SEM of triplicate measurements in milli-P (mP) units.

### GFP-FRET *in vivo* interaction

Single-cell GFP-FRET was measured in a fluorescence imaging system. Excitation of donor (CFP) and acceptor (YFP) fluorescence was at 430 nm and 520 nm, respectively. After baseline recording, the acceptor fluorescence was selectively photobleached by 30 cycles of 5 s expositions at 520 nm while measuring the donor and acceptor fluorescences. Both channels were normalized to the emission intensity immediately before acceptor bleaching. (See Methods S1 for details).

### Electrophysiology

Patch clamp recordings were performed on HEK293 cells in the whole cell configuration. Cells were clamped at a potential of −20 mV, and current-voltage (I–V) relations were obtained from voltage ramps from −100 mV to +100 mV. The standard extracellular solution contained 140 mM NaCl, 5 mM CsCl, 2 mM CaCl_2_, 1 mM MgCl_2_, 10 mM glucose, and 10 mM HEPES (pH 7.4 with NaOH). In nominally Ca^2+^-free solutions, Ca^2+^ was omitted. The standard intracellular solution contained 110 mM cesium methanesulfonate, 25 mM CsCl, 2 mM MgCl_2_, 0.362 mM CaCl_2_, 1 mM EGTA, and 30 mM HEPES (pH 7.2 with CsOH) with a calculated [Ca^2+^] of 100 nM. (See Methods S1 for details).

## Supporting Information

Methods S1 Supplementary Methods.
(0.04 MB DOC)

Figure S1 AlphaScreen-based CaM interaction assay. Streptavidin-coated AlphaScreen donor- and anti-Flag-coated acceptor beads were incubated with the indicated concentrations of CaM-biotin and 6 nM C2-Flag (A) or C2-Flag and 10 nM CaM-biotin (B). Data points show mean±SEM of measurements in buffer containing 100 µM Ca^2+^ (filled squares) or 1 mM EGTA (open squares). The AlphaScreen output signal is shown in arbitrary units (kilo counts per second, kcps).
(0.07 MB TIF)

Figure S2 Ca^2+^-dependent binding of C2 to CaM or CaM mutants that are partially Ca^2+^-binding defective. The Ca^2+^-dependence of C2-biotin binding to wild type CaM-Flag or mutants that are Ca^2+^-binding deficient in the N lobe (CaM_12_), the C lobe (CaM_34_) or both lobes (CaM_1234_) was measured in an AlphaScreen assay at the indicated Ca^2+^ concentrations. Half maximal binding was observed at 8.8±1.0 µM, 10.7±1.0 µM, 27.0±1.1 µM Ca^2+^ for CaM, CaM_12_ and CaM_34_, respectively with a common hill coefficient of 2.0 (n = 3 independent experiments in triplicate). CaM_1234_ did not show measurable C2 interaction. To demonstrate the Ca^2+^-independence of the assay system itself, a GST-Flag-biotin fusion protein was incubated with the AlphaScreen beads at 100 µM Ca^2+^ or 1 mM EGTA (inset). The AlphaScreen readout was independent of the Ca^2+^ concentration.
(0.07 MB TIF)

Figure S3 Identification of CaM binding and interdomain interaction sites in TRPV4. (A) A library of 20-mer peptides overlapping by 18 amino acids and covering the TRPV4 N terminus was spotted on filter paper and probed with biotinylated CaM or C2 at 100 µM Ca^2+^. The peptide dots are shown at the start of the respective 20-mer peptide within the TRPV4 sequence graph. Thus, the underlying peptide sequence actually extends 20 amino acids, corresponding to 10 dots, to the right. The positions of the ankyrin domains, the fragments N2 and N3 and peptides P1, P2 and P3 are indicated. (B) Peptide library of the TRPV4 C terminus, probed with biotinylated CaM or N2 fragment. The positions of the C2-fragment and peptides P4 and P5 are indicated.
(0.31 MBTIF)

Figure S4 CaM binding in the CaM interaction sites in TRPV4. (A–E), In fluorescence polarization experiments, the carboxyfluorescein-labeled peptides P1 (A), P2 (B), P3 (C), P4 (D) and P5 (E) were incubated with CaM, CaM mutants or isolated CaM lobes (see Fig. 1B in main text) at concentrations between 0.1 nM and 10 µM. The respective EC_50_ values are given in Table S2.
(0.11 MB TIF)

Figure S5 The N2 fragment forms dimers in gel filtration experiments. (A) N2 results in a monophasic elution profile with an apparent molecular size that corresponds with a dimeric form of the protein. (B) Calibration was done with a commercial protein mixture with the indicated molecular weights.
(0.07 MB TIF)

Table S1 Positions of the TRPV4 fragments used in the study.
(0.03 MB DOC)

Table S2 CaM binding properties of the putative CaM interaction peptides.
(0.03 MB DOC)
