# Supplementary material for: Interdomain Interactions Control Ca2+-Dependent Potentiation in the Cation Channel TRPV4
Source: PLoS One. 2010 May 11;5(5):e10580. doi: 10.1371/journal.pone.0010580 (PMC2867956; doi:10.1371/journal.pone.0010580)
Supplement: Methods S1 — Supplementary Methods. (0.04 MB DOC) [file pone.0010580.s001.doc]

# Supplementary Methods

### Protein expression and biotinylation

The expression plasmids containing the cDNA for CaM or TRPV4 fragments in the pGEX-2TK vector (Amersham) or a modified derivative that contains an in-frame Flag epitope 5’ to the multiple cloning site were transformed into the BL21(DE3) *Escherichia coli* host, and protein expression was induced by 200 mg/ml IPTG at an OD600 of 0.6. The cells were harvested 3 h after induction, lysed by sonification and the GST fusion proteins extracted from the supernatant by glutathione sepharose (Amersham) affinity chromatography according to the manufacturer’s protocol. The GST-Flag fusion proteins were eluted with 50 mM Tris, pH 8.5, 100 mM KCl, and 10 mM glutathione. The GST-fusion proteins intended for subsequent biotinylation were subjected to an additional wash step with 50 mM HEPES, pH 7.4, 100 mM KCl and eluted with 50 mM HEPES, pH 8.5, 100 mM KCl, and 10 mM glutathione. The purity of all proteins was confirmed by SDS-polyacrylamide gel electrophoresis and Coomassie staining, and the protein concentration determined by the method according to Bradford using an albumin concentration standard. Biotinylation was performed using a 10-fold molar excess of biotinamidocaproate N-hydroxy­succinimide ester (Sigma). After incubation at 4 °C for 12 h, the reaction was stopped by addition of a 10-fold molar excess of NH_4_Cl and unbound biotin ester removed by extensive dialysis against 50 mM Tris, pH 7.4, 100 mM KCl.

Proteins used for competition experiments were subjected to gel filtration into the assay buffer using a PD-10 column (Amersham).

### Gel filtration chromatography

Purified proteins or gel filtration standard (BioRad) were loaded onto a Bio-Sil 250 (BioRad) size exclusion chromatography column and eluted with a buffer containing 50 mM sodium phosphate, pH 7.0 and 200 mM KCl. The elution profile was recorded by continuous absorption measurement at 280 nm.

### AlphaScreen-based CaM interaction assay

To establish an improved interaction assay, that allows the quantification of protein interactions on a medium to high throughput scale, a commercially available proximity assay system based on the AlphaScreen (Amplified Luminescent Proximity Homogenous Assay) technology (PerkinElmer) was modified. The assay principle had been originally developed as an enhanced immunoassay and initially termed luminescent oxygen channeling immunoassay (LOCI [1]). The basic principle that underlies this technology is distance-dependent energy transfer between two microscopic beads of a diameter of about 250 nm through an excited oxygen species (Fig. 2). Upon laser excitation at a wavelength of 680 nm, a phthalocyanine photosensitizer compound in the donor bead converts molecular oxygen contained in the buffer environment into the singlet state. After diffusion to the acceptor bead, the energy transferred by the singlet oxygen is converted into chemo­luminescence by a thioxene derivative in the acceptor bead. The formation of multiple singlet oxygen molecules results in signal amplification. The resulting emission at a wavelength of 370 nm is further transformed by fluorophores within the bead into a final emission between 520 nm and 620 nm that can be detected by a photomultiplier. As singlet oxygen has a lifespan of around 4 µs in aqueous solutions, its diffusion distance is limited to around 200 nm. Consequently, the output signal is strongly dependent on the distance between the beads and can therefore be used to measure the interaction of substances bound to the surface of the beads. In this study, streptavidin-coated donor beads were used to immobilize a biotinylated protein component, CaM or CaM-binding proteins, and anti-Flag antibody bound to the acceptor bead surface was used to hold the Flag epitope-tagged interacting protein component. All interaction experiments were done with proteins heterologously expressed in *E. coli* and purified by affinity chromatography.

As the actual protein-binding capacity of the donor and acceptor beads can not readily be predicted, optimal component concentrations were empirically determined by titration. 10 nM CaM-biotin and TRPV4-C2 concentrations ranging between 0.1 nM and 1 µM or 6 nM TRPV4-C2 and CaM-biotin concentrations between 0.1 nM and 1 µM were used in the described AlphaScreen assay (Fig. S1A,B). Measurements were done in buffer containing either 100 µM Ca^2+^ or 1 mM EGTA. The recording of the AlphaScreen output signals showed a bell-shaped dependence on the C2-Flag and CaM-biotin concentrations at 100 µM Ca^2+^. The strongest signal was obtained at 12 nM C2-Flag and 19 nM CaM-biotin. In both experiments, the ascending part of the curve reflects the progressive saturation of the binding sites of the donor and acceptor beads, respectively. As an increasing proportion of the beads are recruited for the interaction, the output signal is enhanced. At concentrations above the binding capacity, free molecules compete with the surface-bound protein and lower the interaction signal. No signal was detected in buffers containing 1 mM EGTA. All further AlphaScreen interaction experiments were performed with binding protein concentrations of 10 nM.

### Peptide library overlay

Libraries of 20-mer peptides that cover the TRPV4 N terminus to the first transmembrane domain and the C terminus starting after the sixth transmembrane domain in increments of two amino acids were in situ synthesized on filter paper (Dr. Sven Rothemund, core unit peptide technologies, IZKF Leipzig). Dried filters were hydrated over night at 4°C in buffer containing 50 mM Tris, pH 7.4, 100 mM NaCl and blocked with 3 % non-fat milk powder for 4 h at room temperature in the same buffer. Protein overlay was done using 100 nM biotinylated CaM-GST, C2-GST or N3-GST in the above buffer. Incubation for 90 min at room temperature was followed by extensive washing with the same buffer containing 0.03 % Tween-20. For the detection of bound protein, the blots were incubated with 0.5 µg/ml peroxidase-coupled avidin (Sigma) for 50 min at room temperature, washed as described and the peroxidase activity visualized by chemoluminescence.

### GFP-FRET interaction in living cells

HEK293 cells were cultured as described previously [2] and plated on glass cover slips 24 h before transfection. Cells were transiently cotransfected with 500 ng each of the donor and acceptor plasmids. The plasmids were constructed from the pcDNA3 vector (Invitrogen) and contained the CFP or YFP coding sequence fused to the 5’-end and the 3’-end of the insert, respectively. FRET was measured on the single-cell level in a microscope-based fluorescence imaging system (TILL Photonics) equipped with an excitation monochromator and an automated emission filter wheel providing D480/40 and D560/40 emission filters (Sutter Instrument).

### Electrophysiology

Patch clamp recordings were performed on HEK293 cells in the whole cell configuration using an EPC10 amplifier and Pulse software (HEKA, Lamprecht, Germany). Patch pipettes were made from borosilicate glass and had resistances of 3 – 5 MΩ when filled with the standard intracellular solution. Cells were clamped at a potential of –20 mV, and current-voltage (I-V) relations were obtained from voltage ramps from –100 mV to +100 mV with a duration of 400 ms applied every 4 seconds. Ramp data were acquired with a sampling rate of 20 kHz after filtering at 4 kHz. The standard extracellular solution contained 140 mM NaCl, 5 mM CsCl, 2 mM CaCl_2_, 1 mM MgCl_2_, 10 mM glucose, and 10 mM HEPES (pH 7.4 with NaOH). In nominally Ca^2+^-free solutions, Ca^2+^ was omitted. Stock solutions of 4α-PMA (Sigma) were made in DMSO and diluted to final concentrations in the bath solutions. The standard intracellular solution contained 110 mM cesium methanesulfonate, 25 mM CsCl, 2 mM MgCl_2_, 0.362 mM CaCl_2_, 1 mM EGTA, and 30 mM HEPES (pH 7.2 with CsOH) with a calculated [Ca^2+^] of 100 nM (MaxChelator, Chris Patton, Stanford, USA). The osmolarity of all solutions was between 290 and 310 mosmol/l. HEK293 cells were maintained as described in the supplier’s recommendations. Transient transfection of the cDNAs of TRPV4-YFP, TRPV4-KRA-YFP or TRPV4-WIA-YFP (1 µg each) was performed using FuGene® HD transfection kit (Roche Molecular Biochemicals) 1 day after seeding cells onto glass coverslips (Menzel, Braunschweig, Germany). In some experiments shown in Fig. 1F, cells were cotransfected with 1 µg of the cDNA of CaM-CFP or mutants thereof. All patch-clamp experiments were performed at room temperature (20 – 25°C) 1 – 2 days after transfection.

# References

## 1. Ullman EF, Kirakossian H, Singh S, Wu ZP, Irvin BR, et al. (1994) Luminescent oxygen channeling immunoassay: measurement of particle binding kinetics by chemiluminescence. Proc Natl Acad Sci U S A 91: 5426-5430.

## 2. Strotmann R, Harteneck C, Nunnenmacher K, Schultz G, Plant TD (2000) OTRPC4, a nonselective cation channel that confers sensitivity to extracellular osmolarity. Nat Cell Biol 2: 695-702.
