# Supplementary figures and images for: Interdomain Interactions Control Ca2+-Dependent Potentiation in the Cation Channel TRPV4
Source: PLoS One. 2010 May 11;5(5):e10580. doi: 10.1371/journal.pone.0010580 (PMC2867956; doi:10.1371/journal.pone.0010580)

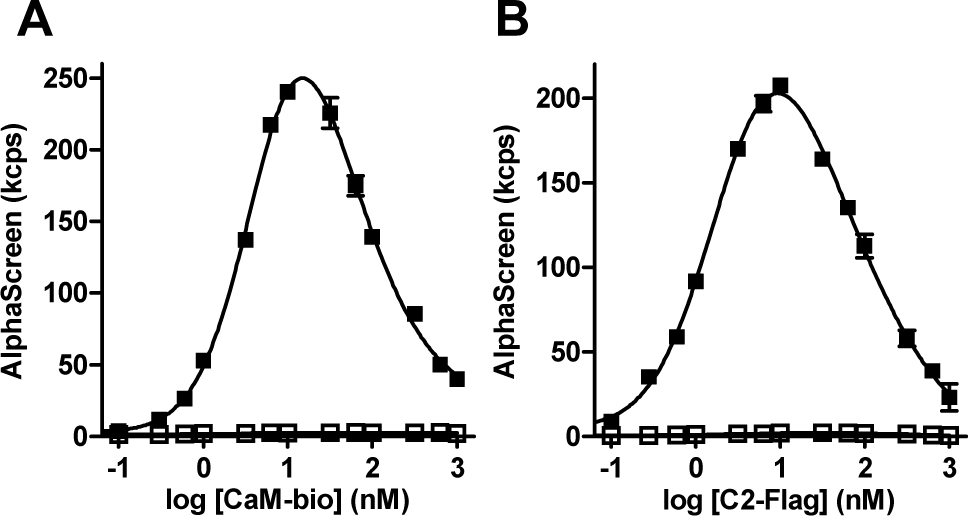

Supplement: Figure S1 — AlphaScreen-based CaM interaction assay. Streptavidin-coated AlphaScreen donor- and anti-Flag-coated acceptor beads were incubated with the indicated concentrations of CaM-biotin and 6 nM C2-Flag (A) or C2-Flag and 10 nM CaM-biotin (B). Data points show mean±SEM of measurements in buffer containing 100 µM Ca2+ (filled squares) or 1 mM EGTA (open squares). The AlphaScreen output signal is shown in arbitrary units (kilo counts per second, kcps). (0.07 MB TIF) [file pone.0010580.s002.tif]

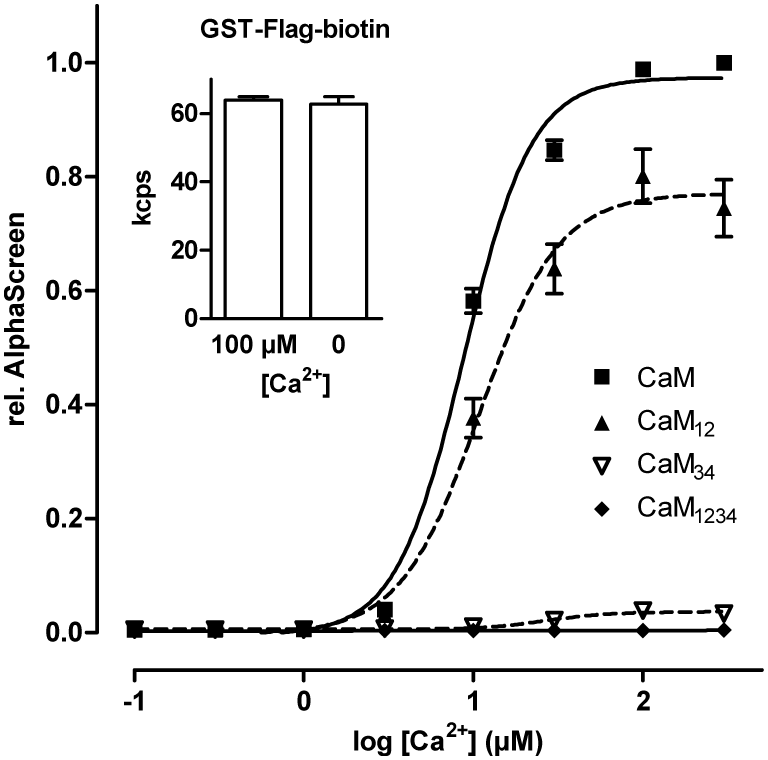

Supplement: Figure S2 — Ca2+-dependent binding of C2 to CaM or CaM mutants that are partially Ca2+-binding defective. The Ca2+-dependence of C2-biotin binding to wild type CaM-Flag or mutants that are Ca2+-binding deficient in the N lobe (CaM12), the C lobe (CaM34) or both lobes (CaM1234) was measured in an AlphaScreen assay at the indicated Ca2+ concentrations. Half maximal binding was observed at 8.8±1.0 µM, 10.7±1.0 µM, 27.0±1.1 µM Ca2+ for CaM, CaM12 and CaM34, respectively with a common hill coefficient of 2.0 (n = 3 independent experiments in triplicate). CaM1234 did not show measurable C2 interaction. To demonstrate the Ca2+-independence of the assay system itself, a GST-Flag-biotin fusion protein was incubated with the AlphaScreen beads at 100 µM Ca2+ or 1 mM EGTA (inset). The AlphaScreen readout was independent of the Ca2+ concentration. (0.07 MB TIF) [file pone.0010580.s003.tif]

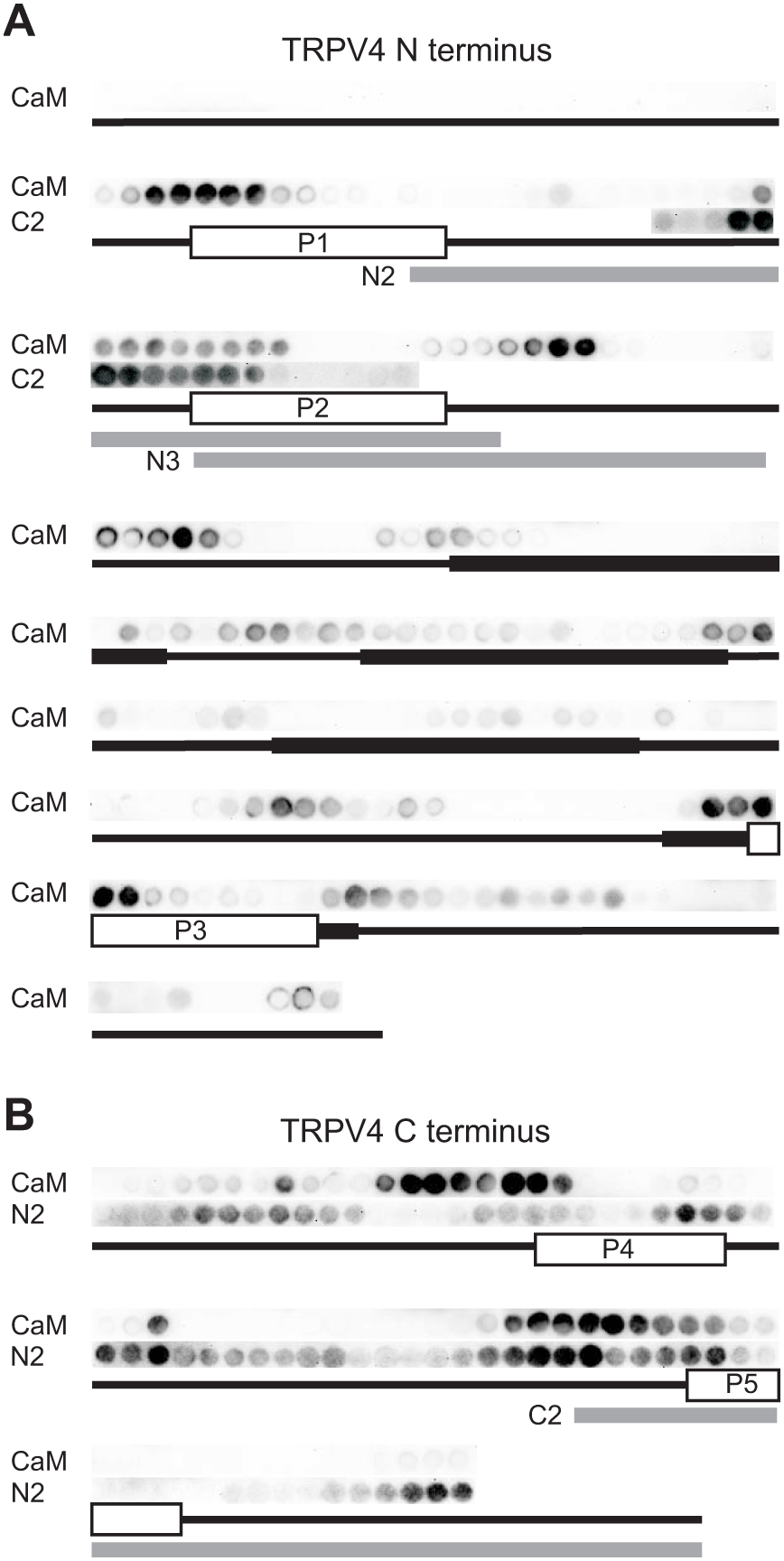

Supplement: Figure S3 — Identification of CaM binding and interdomain interaction sites in TRPV4. (A) A library of 20-mer peptides overlapping by 18 amino acids and covering the TRPV4 N terminus was spotted on filter paper and probed with biotinylated CaM or C2 at 100 µM Ca2+. The peptide dots are shown at the start of the respective 20-mer peptide within the TRPV4 sequence graph. Thus, the underlying peptide sequence actually extends 20 amino acids, corresponding to 10 dots, to the right. The positions of the ankyrin domains, the fragments N2 and N3 and peptides P1, P2 and P3 are indicated. (B) Peptide library of the TRPV4 C terminus, probed with biotinylated CaM or N2 fragment. The positions of the C2-fragment and peptides P4 and P5 are indicated. (0.31 MBTIF) [file pone.0010580.s004.tif]

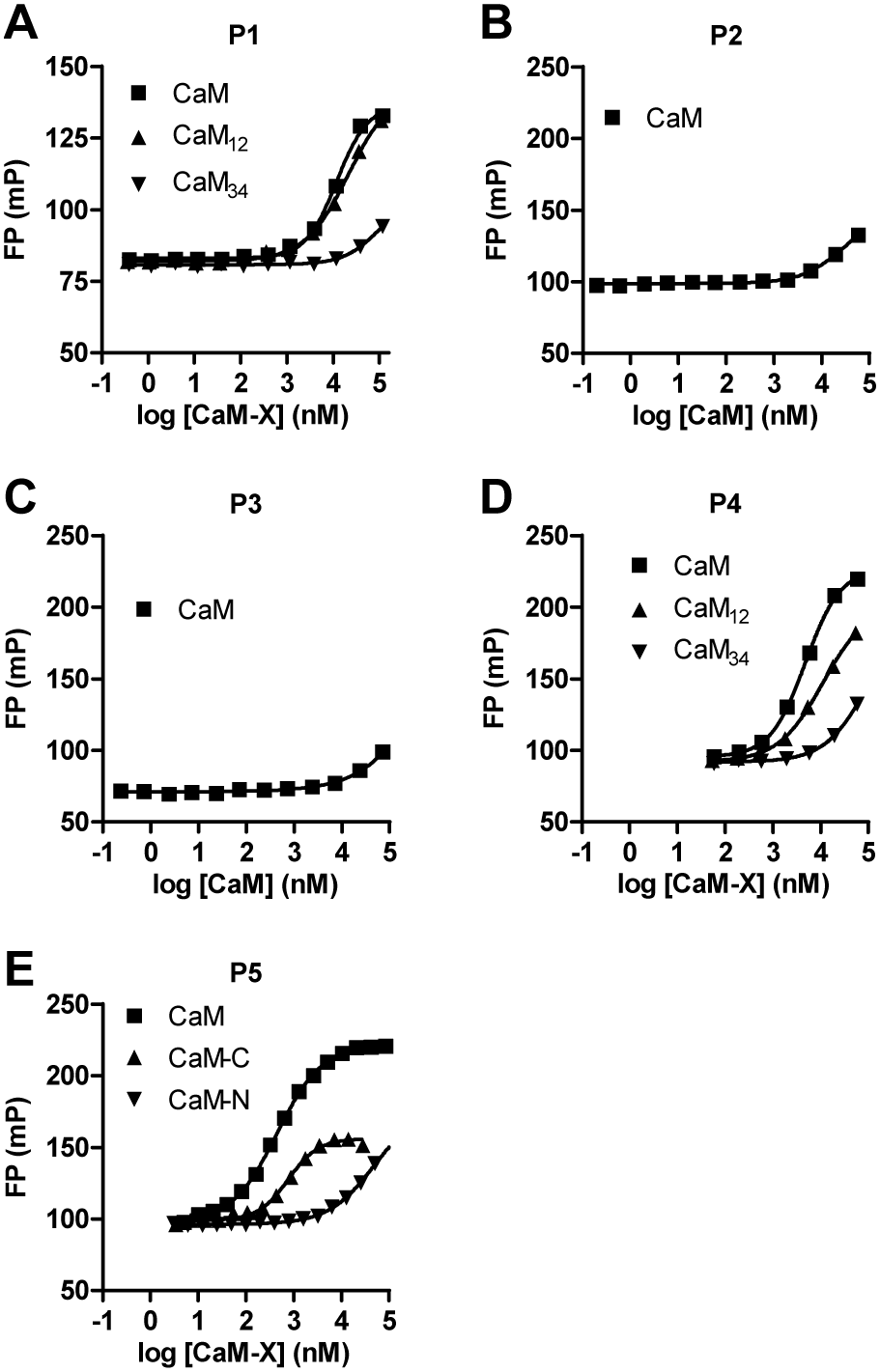

Supplement: Figure S4 — CaM binding in the CaM interaction sites in TRPV4. (A–E), In fluorescence polarization experiments, the carboxyfluorescein-labeled peptides P1 (A), P2 (B), P3 (C), P4 (D) and P5 (E) were incubated with CaM, CaM mutants or isolated CaM lobes (see Fig. 1B in main text) at concentrations between 0.1 nM and 10 µM. The respective EC50 values are given in Table S2. (0.11 MB TIF) [file pone.0010580.s005.tif]

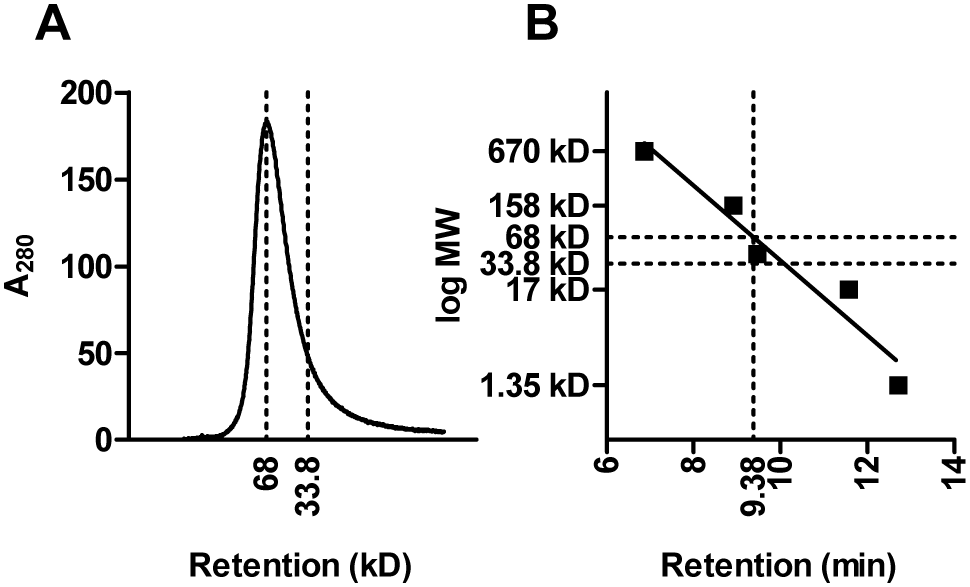

Supplement: Figure S5 — The N2 fragment forms dimers in gel filtration experiments. (A) N2 results in a monophasic elution profile with an apparent molecular size that corresponds with a dimeric form of the protein. (B) Calibration was done with a commercial protein mixture with the indicated molecular weights. (0.07 MB TIF) [file pone.0010580.s006.tif]
