# Supplementary material for: Interdomain Interactions Control Ca2+-Dependent Potentiation in the Cation Channel TRPV4
Source: PLoS One. 2010 May 11;5(5):e10580. doi: 10.1371/journal.pone.0010580 (PMC2867956; doi:10.1371/journal.pone.0010580)
Supplement: Table S1 — Positions of the TRPV4 fragments used in the study. (0.03 MB DOC) [file pone.0010580.s007.doc]

| **Peptide** | **Start** | **End** | **Sequence (N to C terminus)** |
| --- | --- | --- | --- |
| N1 | 1 | 471 | MADSS ... GAVSF |
| N2 | 79 | 138 | VPNPI ... SPKAP |
| N3 | 114 | 161 | RHHSS ... IVSRG |
| C1 | 721 | 871 | TVGQV ... EDAPL |
| C2 | 806 | 871 | QYYGF ... EDAPL |
| P1 | 63 | 82 | GRPNLRMKFQGAFRKGVPNP |
| P2 | 117 | 136 | SSDNKRWRKKIIEKQPQSPK |
| P3 | 377 | 396 | AAKTGKIGIFQHIIRREVTD |
| P4 | 753 | 776 | RSFPVFLRKAFRSGEMVTVGKSSD |
| P5 | 816 | 838 | GFSHTVGRLRRDRWSSVVPRVVE |
