# Supplementary material for: Interdomain Interactions Control Ca2+-Dependent Potentiation in the Cation Channel TRPV4
Source: PLoS One. 2010 May 11;5(5):e10580. doi: 10.1371/journal.pone.0010580 (PMC2867956; doi:10.1371/journal.pone.0010580)
Supplement: Table S2 — CaM binding properties of the putative CaM interaction peptides. (0.03 MB DOC) [file pone.0010580.s008.doc]

| **Peptide** | **CaM binding EC_50_** | **Ca^2+^-dependence, EC_50_^#^** | **CaM lobe binding preference** |
| --- | --- | --- | --- |
| P1 | 11 µM | 3.6 µM | CaM = CaM_12_ > CaM_34_ |
| P2 | ~ 25 µM | nd | nd |
| P3 | > 100 µM | nd | nd |
| P4 | 5 µM | 7.3 µM | CaM > CaM_12_ > CaM_34_ |
| P5 | 450 nM | 3.2 µM | CaM > CaM-C > CaM-N |

nd: not determined
^#^Ca^2+^ concentration at half-maximal binding
